# Supplementary material for: Process- and product-related impurities in the ChAdOx1 nCov-19 vaccine
Source: eLife. 2022 Jul 4;11:e78513. doi: 10.7554/eLife.78513 (PMC9313527; doi:10.7554/eLife.78513)
Supplement: Figure 1—source data 1. [file elife-78513-fig1-data1.pdf]

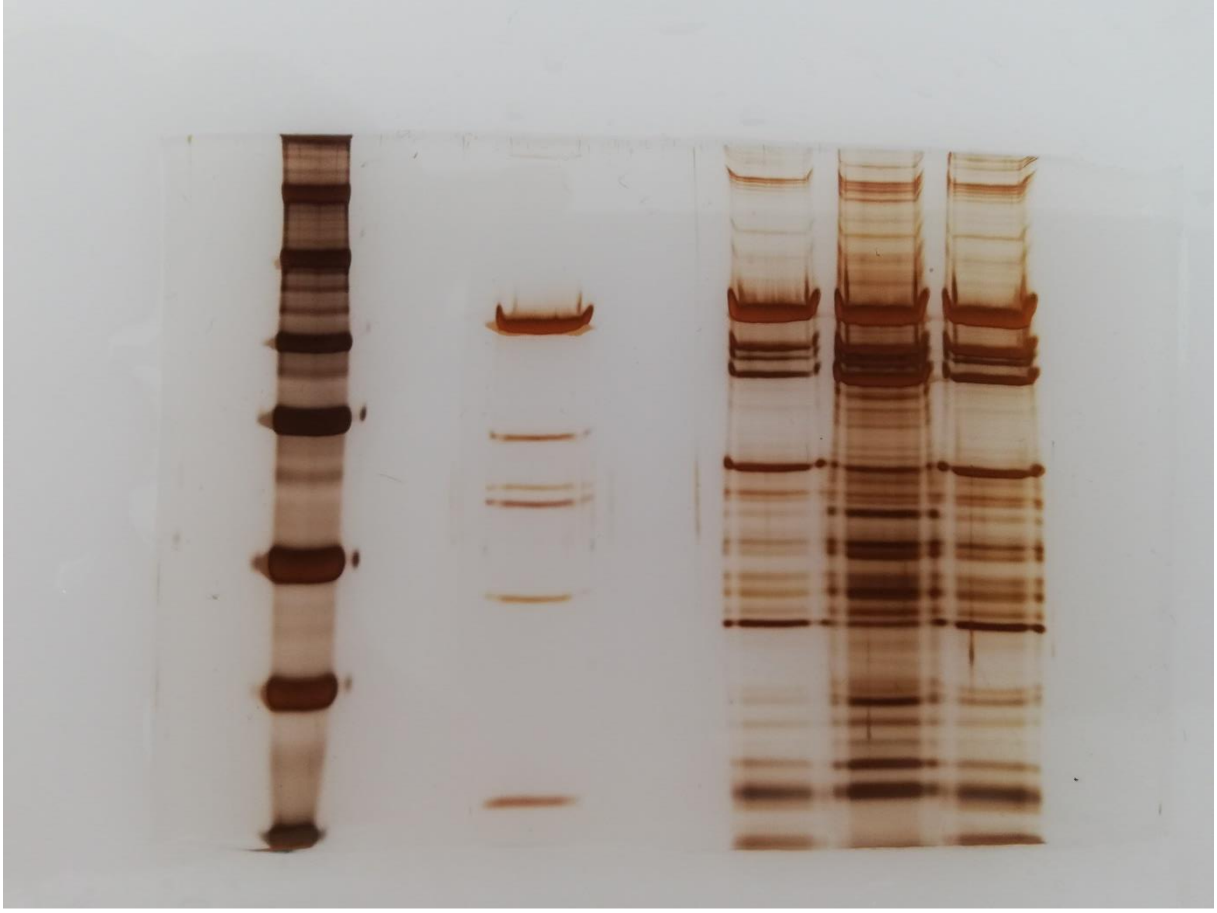

Figure 1 – source data 1_Original file of the full raw unedited gel: Protein staining of HAdV-C5-EGFP and three ChAdOx1 nCoV-19 vaccine lots. 3 x 10^9 adenoviral vector particles were separated by SDS-PAGE under denaturing and reducing conditions. Proteins were visualized by silver staining. Lane 1: Reference protein marker; lane 2: HAdV-5-EGFP; lane 3-5: three different vaccine lots (ABV4678, ABV5811, ABV7764) of ChAdOx1, produced by the manufacturer.
