# Supplementary material for: Process- and product-related impurities in the ChAdOx1 nCov-19 vaccine
Source: eLife. 2022 Jul 4;11:e78513. doi: 10.7554/eLife.78513 (PMC9313527; doi:10.7554/eLife.78513)
Supplement: Figure 1—source data 2. [file elife-78513-fig1-data2.pdf]

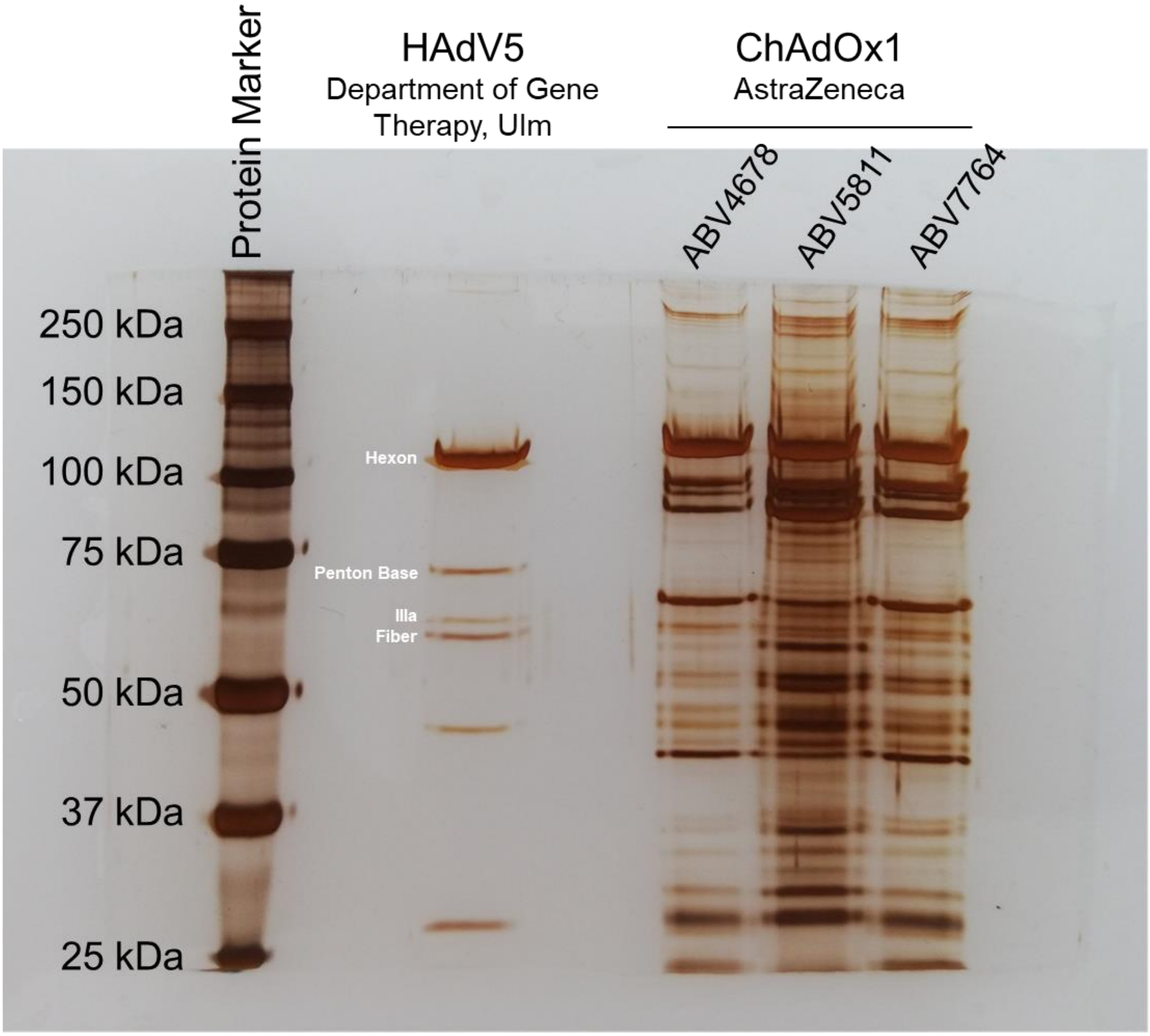

Figure 1 – source data 2_ Uncropped gel with the relevant bands labeled: Protein staining of HAdV-C5-EGFP and three ChAdOx1 nCoV-19 vaccine lots. 3×10^9 adenoviral vector particles were separated by SDS-PAGE under denaturing and reducing conditions. Proteins were visualized by silver staining. Known HAdV-C5 proteins are labeled. Marker bands are labeled. Three different vaccine lots (ABV4678, ABV5811, ABV7764) of ChAdOx1, produced by the manufacturer, were analyzed. kDa: kilodalton
