# Supplementary material for: Process- and product-related impurities in the ChAdOx1 nCov-19 vaccine
Source: eLife. 2022 Jul 4;11:e78513. doi: 10.7554/eLife.78513 (PMC9313527; doi:10.7554/eLife.78513)
Supplement: Figure 3—source data 2. [file elife-78513-fig3-data2.pdf]

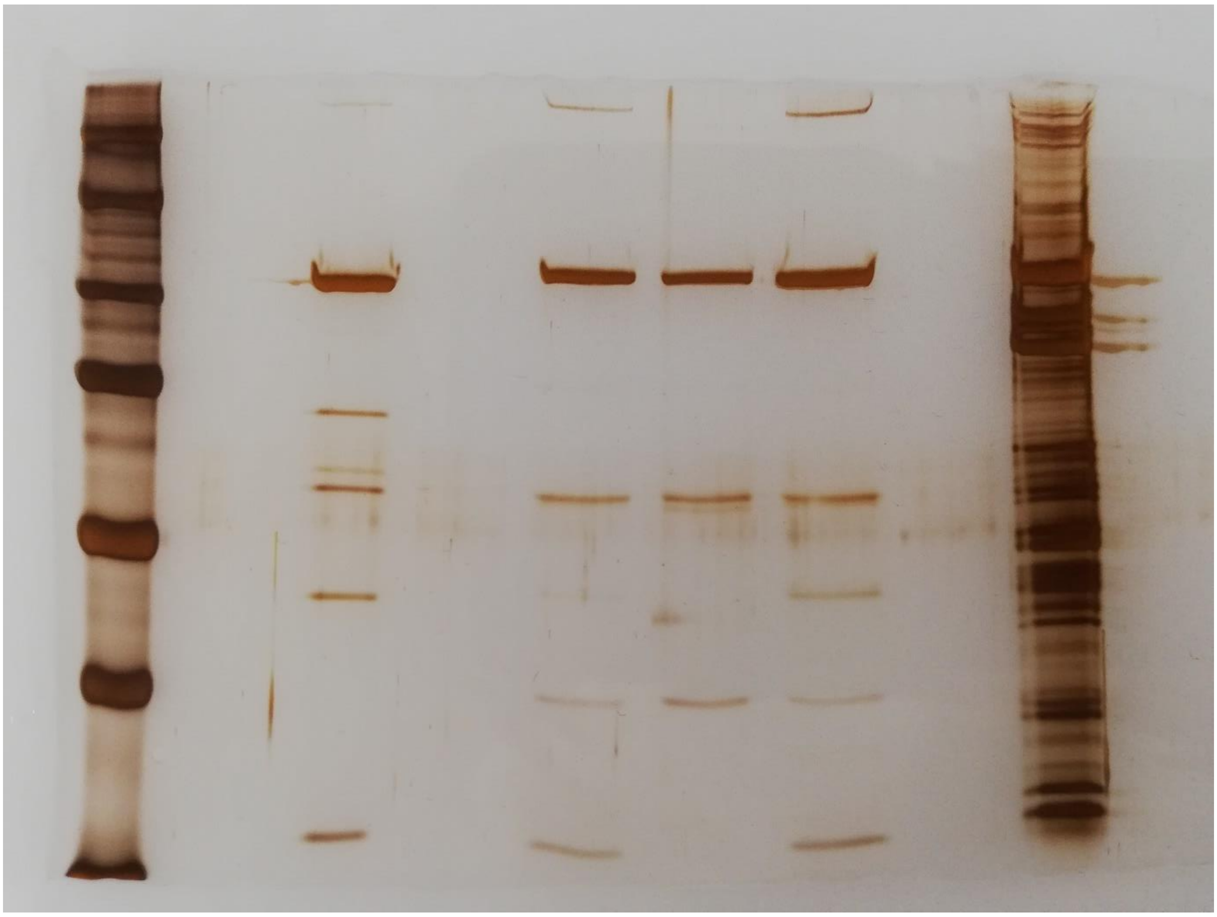

Figure 3A – source data 1_Original file of the full raw unedited gel: Comparison of Ad26.COV2.S and ChAdOx1 nCov-19 vaccines. 3 x 10^9 adenoviral vector particles were separated by SDS-PAGE under denaturing and reducing conditions. Proteins were visualized by silver staining. Lane 1: Reference protein marker; lane 2: HAdV-5-EGFP; lane 3-5: three lots of Ad26.COV2.S (21C10-01, XD955, XE395); lane 6: one lot of ChAdOx1 (ABV9317).
