# Supplementary material for: Process- and product-related impurities in the ChAdOx1 nCov-19 vaccine
Source: eLife. 2022 Jul 4;11:e78513. doi: 10.7554/eLife.78513 (PMC9313527; doi:10.7554/eLife.78513)
Supplement: Figure 3—source data 3. [file elife-78513-fig3-data3.pdf]

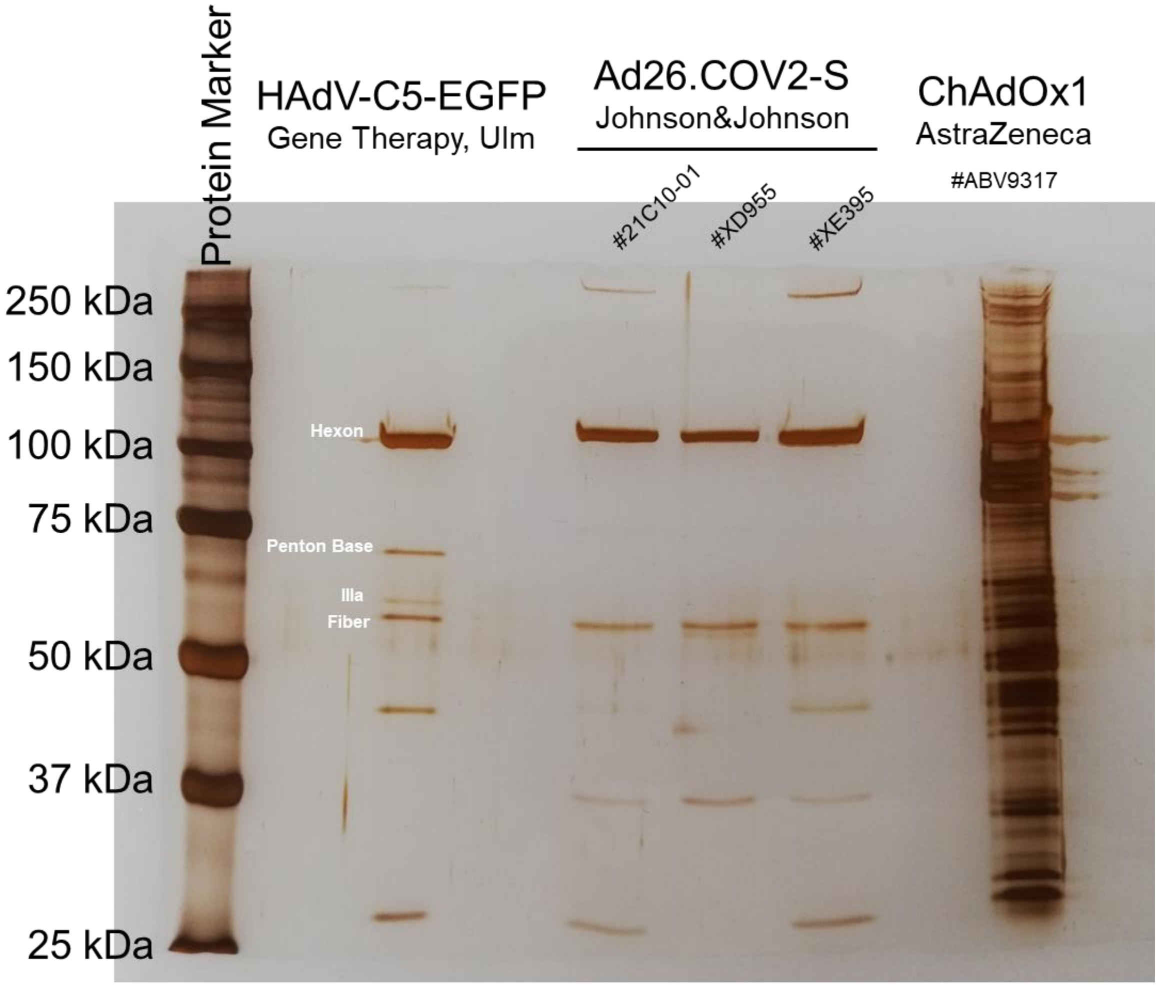

Figure 3A – source data 2_ Uncropped gel with the relevant bands labeled: Protein staining of HAdV-C5-EGFP, three Johnson&Johnson Ad25-COV2.S vaccine lots and one AstraZeneca ChAdOx1 nCoV-19 vaccine lot. 3 x 10^9 adenoviral vector particles were separated by SDS-PAGE under denaturing and reducing conditions. Proteins were visualized by silver staining. Known HAdV-C5 proteins are labeled. Marker bands are labeled. Three lots of Ad26.COV2.S (21C10-01, XD955, XE395) and one lot of ChAdOx1 (ABV9317).
