# Supplementary material for: Process- and product-related impurities in the ChAdOx1 nCov-19 vaccine
Source: eLife. 2022 Jul 4;11:e78513. doi: 10.7554/eLife.78513 (PMC9313527; doi:10.7554/eLife.78513)
Supplement: Figure 4—source data 2. [file elife-78513-fig4-data2.pdf]

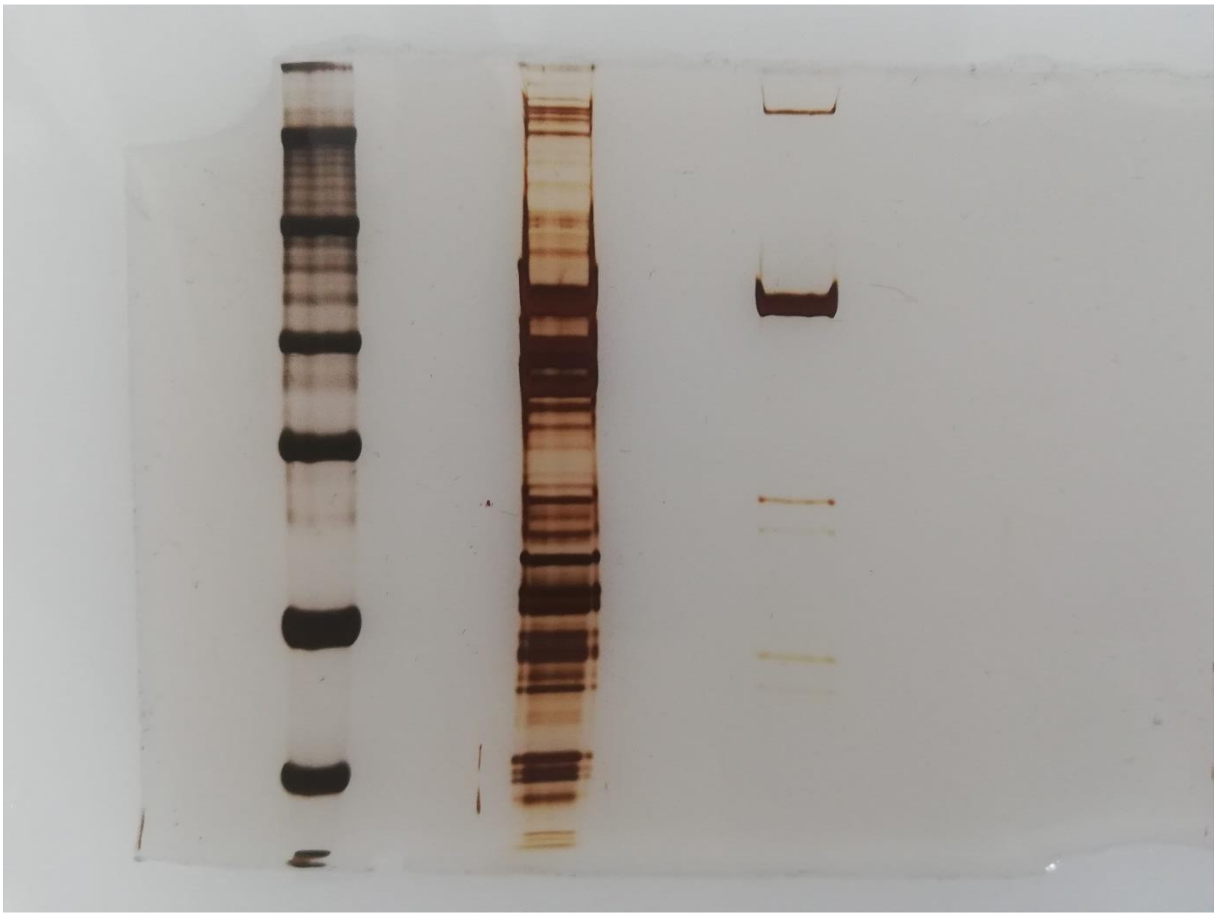

Figure 4A – source data 1_Original file of the full raw unedited gel: Protein staining of ChAdOx1 nCoV-19 vaccine lot and UUlm ChAdOx1. 3 x 10^9 adenoviral vector particles were separated by SDS-PAGE under denaturing and reducing conditions. Proteins were visualized by silver staining. Lane 1: Reference protein marker; lane 2: AstraZeneca ChAdOx1 vaccine lot ABV9317 produced by the manufacturer; lane 3: CsCl-purified UUlm ChAdOx1.
