# Supplementary material for: Process- and product-related impurities in the ChAdOx1 nCov-19 vaccine
Source: eLife. 2022 Jul 4;11:e78513. doi: 10.7554/eLife.78513 (PMC9313527; doi:10.7554/eLife.78513)
Supplement: Figure 4—source data 3. [file elife-78513-fig4-data3.pdf]

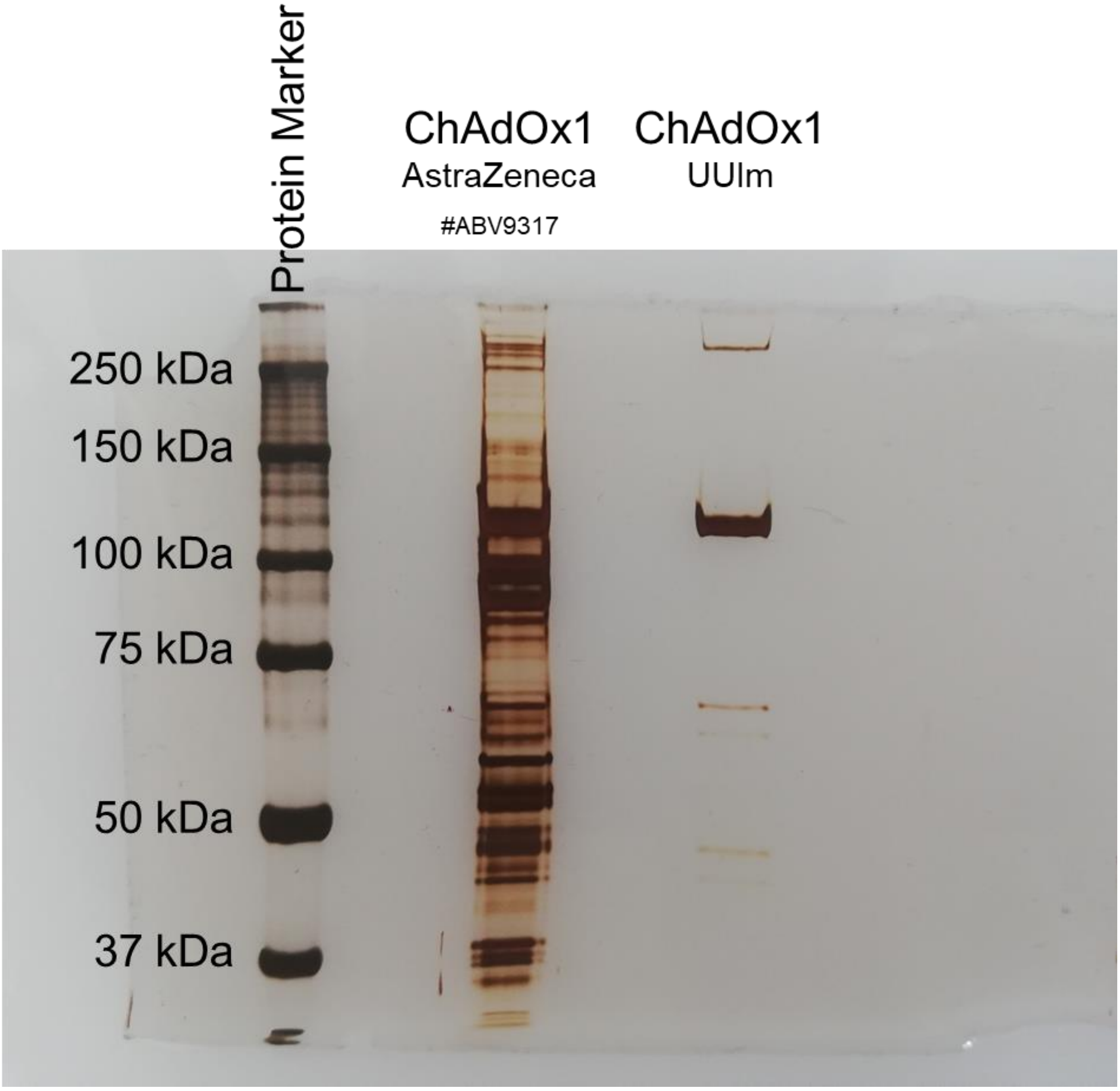

Figure 4A – source data 2_ Uncropped gel with the relevant bands labeled: Protein staining of ChAdOx1 nCoV-19 vaccine lot and UUlm ChAdOx1. 3 x 10^9 adenoviral vector particles were separated by SDS-PAGE under denaturing and reducing conditions. Proteins were visualized by silver staining. Marker bands are labeled. ChAdOx1 ABV9317 was purified by the manufacturer; UUlm ChAdOx1 was purified by CsCl density gradient centrifugation.
